# Fpr2 exacerbates *Streptococcus suis*-induced streptococcal toxic shock-like syndrome *via* attenuation of neutrophil recruitment

**DOI:** 10.3389/fimmu.2023.1094331

**Published:** 2023-01-27

**Authors:** Chengpei Ni, Song Gao, Xudong Li, Yuling Zheng, Hua Jiang, Peng Liu, Qingyu Lv, Wenhua Huang, Qian Li, Yuhao Ren, Zhiqiang Mi, Decong Kong, Yongqiang Jiang

**Affiliations:** ^1^ School of Basic Medical Sciences, Anhui Medical University, Hefei, China; ^2^ State Key Laboratory of Pathogen and Biosecurity, Institute of Microbiology and Epidemiology, Academy of Military Medical Sciences, Beijing, China; ^3^ The Affiliated Wuxi Center for Disease Control and Prevention of Nanjing Medical University, Wuxi Center for Disease Control and Prevention, Wuxi, China

**Keywords:** *Streptococcus suis*, leukocyte recruitment, NETs, formyl peptide receptor 2, STSLS

## Abstract

The life-threatening disease streptococcal toxic shock-like syndrome (STSLS), caused by the bacterial pathogen *Streptococcus suis* (*S. suis*). Proinflammatory markers, bacterial load, granulocyte recruitment, and neutrophil extracellular traps (NETs) levels were monitored in wild-type (WT) and Fpr2^-/-^ mice suffering from STSLS. LXA4 and AnxA1, anti-inflammatory mediators related to Fpr2, were used to identity a potential role of the Fpr2 in STSLS development. We also elucidated the function of Fpr2 at different infection sites by comparing the STSLS model with the *S. suis*-meningitis model. Compared with the WT mice, Fpr2^-/-^ mice exhibited a reduced inflammatory response and bacterial load, and increased neutrophil recruitment. Pretreatment with AnxA1 or LXA4 impaired leukocyte recruitment and increased both bacterial load and inflammatory reactions in WT but not Fpr2^-/-^ mice experiencing STSLS. These results indicated that Fpr2 impairs neutrophil recruitment during STSLS, and this impairment is enhanced by AnxA1 or LXA4. By comparing the functions of Fpr2 in different *S. suis* infection models, inflammation and NETs was found to hinder bacterial clearance in *S. suis* meningitis, and conversely accelerate bacterial clearance in STSLS. Therefore, interference with neutrophil recruitment could potentially be harnessed to develop new treatments for this infectious disease.

## Introduction

Infection by the major pathogenic gram-positive bacterium *Streptococcus suis* can cause life-threatening meningitis and sepsis in humans and in swine, which can lead to significant economic losses by the pig breeding industry. The severe lethality of two recent outbreaks in humans was attributed to invasive multiple organ failure resulting from streptococcal toxic-shock-like syndrome (STSLS) (1, 2). Previous studies have indicated that Suilysin, a pore-forming cholesterol-dependent cytolysin of *S. suis*, could activate the inflammasome and cause STSLS (3–5). Although STSLS was long time considered to result from excessive activation of the inflammatory response, the inefficacy of anti-cytokine therapy in several clinical trials determined that the pathogenesis of sepsis was complex. Current research suggests that a proinflammatory response and an anti-inflammatory response occur concurrently in septic patients (6). Because of the ineffectiveness of current treatments, research focusing on the development of therapeutics for STSLS is urgent.

Fpr2 is an intriguing G‐protein‐coupled chemoattractant receptor (GPCR) known for its dual role in immunoregulation (7). It exerts pro-resolution properties by binding anti-inflammatory mediators such as annexin A1(AnxA1) and lipoxin A4 (LXA4), which has been confirmed in both *in vitro* and *in vivo* experiments (8). However, Fpr2 also possesses the ability to sense formyl peptides or serum amyloid A (SAA) to induce pro-inflammatory responses (9). Due to the diversity of Fpr2 ligands, the role of Fpr2 in host immune response depends on the stages of infection and associated ligand profile. The dual roles of Fpr2 make its function in pathogenesis difficult to determine. Although Fpr2 is widely distributed across multiple cells and tissues, it is mainly expressed in myeloid cells. The mouse Fpr2 is most similar to human Fpr2 structurally and functionally, as confirmed by *in vivo* and *in vitro* research (10).

As the most abundant granulocytes, neutrophils serve as the first line of defense against invading pathogens *via* their functions in phagocytosis, and reactive oxygen species (ROS) and protease production. Additionally, neutrophil extracellular traps (NETs), formulated by active neutrophils under certain conditions, have recently been identified as a novel bactericidal mechanism. NETs contain a web of extracellular DNA, histones, myeloperoxidase, and elastases, which strengthen their antimicrobial effect (11). NETs are highly effective at killing bacteria and hindering their spread. Although excessive activation of NETs in sepsis is associated with multiple organ failure, NETs undoubtedly play protective roles in the initial stages of infection. The role of Fpr2 in regulating NETs formation in active neutrophils when stimulated with methicillin-resistant *Staphylococcus aureus* (MRSA) is thought to be associated with an endogenous anti-inflammatory ligand of Fpr2 in the lipoxin pathway (12). The impact of neutrophil recruitment on disease development is dependent on disease pathology, genetics, and environmental stimuli.

Fpr2 impairs the neutrophil recruitment during infection, and AnxA1 has a protective effect in downregulating inflammation through Fpr2 (13). As the immunological responses to bacterial meningitis and STSLS may be quite different, we aimed to investigate the role of Fpr2 in *S. suis*-induced systemic infection, concentrating on the effects of the pro-resolution agents AnxA1 and LXA1 on STSLS progression. By comparing the *S. suis*-meningitis model to the STSLS models, we were able to identify differences in the immune regulation mechanism of Fpr2 in these different *S. suis* infection models. We demonstrated a regulatory role of Fpr2 in controlling neutrophil recruitment, which contributes to the pathogenesis of STSLS pathogenesis by decreasing neutrophil recruitment and NETs formation during the early stages of infection. We also found that inhibition of neutrophil recruitment and NETs formation produced opposing effects on disease progression between bacterial meningitis and systemic infection. This study provides valuable fundamental information for further research into treatment of *S. suis* infection and potentially other infectious diseases.

## Materials and methods

### Bacteria and animals

The wild-type strain *S. suis* 05ZYH33 is a clinical isolate that caused an STSLS outbreak in Sichuan Province of China. *S. suis* was grown in Todd–Hewitt Broth (THB) medium at 37°C and were harvested for experimentation at the mid-log growth phase bacteria. 6 to 8 weeks old C57BL/6J female mice were purchased from SPF (Beijing, China) Biotechnology Co., Ltd. C57BL/6J Fpr2 ^-/-^ mice was generated as indicated in previous studies (13). In brief, Fpr2-deficient mice were generated using the Cre/LoxP system. Mice harboring a floxed allele of Fpr2 (*Fpr2^loxP^/^+^*) were obtained from Cyagen Biosciences (Guangzhou, China); these mice were interbred to generate Fpr2*^loxP/loxP^* mice. Fpr2*^loxP/loxP^* mice were then mated with EIIa-cre transgenic mice to generate a null allele of Fpr2 (Fpr2*^+/-^*). Fpr2*^+/-^* mice were identified by PCR genotyping using multiple primers (mFpr2_F1 [CTCATACGCATTTGCTGTCTTCACAC], mFpr2_R1 [TCCAATTATATCCCTTTCATGGCAAAC], and mFpr2_F3 [ACAAGGGCCTGCATGTGCCCTCTG]). Finally, Fpr2*^+/-^* mice were interbred to generate Fpr2^-/-^ mice.

### Infection

For the STSLS model, a standard bacterial dose (2 × 10^7^ CFU) of bacterial suspensions or a vehicle solution (THB) were infused intraperitoneally in WT or Fpr2^-/-^ mice. In survival experiments, high dose (5 × 10^7^ CFU) of *S. suis* were used. Peritoneal lavage fluid (PLF), blood, and tissues were obtained for analysis at the indicated time point during infection. Bacterial viability was monitored by plating serial dilutions of blood or PLF. Cytokine and chemokine levels and hematoxylin and eosin (H&E) stain were also performed at 12 h post-infection. All the mice were monitored up to 5 d and clinical score were assigned according to the scoring criteria developed in a previous study (14): 4 = dead; 3 = non-responsive or walking in circles; 2 = responds only to repeated stimuli; 1 = ruffled coat and slow response to stimuli, and 0 = normal response to stimuli. For the meningitis model, mice were inoculated with 10 ul of bacterial suspension (1.25 × 10^5^ CFU) suspension intracisternally after general anesthesia (pentobarbital sodium, 50 mg/kg). Mice were monitored up to 72 h to record deaths. Bacterial load in the brain and blood were evaluated at 14 h, when most animals developed clinical symptoms.

### Antagonist

For the interference test, Fpr2/ALX antagonist, WRW4 (Sangon, China) and BOC-2 (N-t-Boc-Phe-Leu-Phe-Leu-Phe; Sangon, China,10 μg/kg; i.p.) were administered after 1 h of infection. In the remaining experiments, animals were treated with AnxA1 (Biobry, UK, 50 μg/kg; i.p.) or LXA4 (Cayman Chemical Company, USA; 2.5 μg/kg; i.p.) 1 h after infection (15).

### Neutrophil-depleting

26 mg/kg of anti-mouse Ly6G (BioXcell, USA, clone:1A8), InVivoMAb, or IgG2a isotype control (BioXcell, USA, clone:2A3) were injected into mice intraperitoneally (i.p.) before infection (16).

### FACS analysis

To determine the activity of neutrophils or macrophage recruitment, the PLF of mice was collected and analysis by FACS. At each indicated time point, animals were euthanized by carbon dioxide asphyxiation, and the abdomen gently massaged. 10 ml sterile harvest solution (PBS+EDTA) was injected intraperitoneally, then the peritoneal fluid was withdrawn and centrifuged (10 min, 200 × g) to collect recruited leukocytes. After quantifying the absolute number, cells were treated with an FcR-blocking reagent (CD16/CD32, BD Pharmingen) for 15 min, then cells incubated with mixed antibodies: FITC-conjugated anti-mouse CD45 (N418, Biolegend, USA); PerCP-Cy5.5-conjugated anti-mouse CD11b (M1/70 Biolegend, USA) APC-conjµgated anti-mouse F4/80 (BM8, Biolegend, USA), PE-conjugated anti-mouse Ly6G (RB6-8C5 Biolegend, USA), PE-Cy7-conjµgated anti-mouse Ly6C (HK1.4 Biolegend, USA), and APC-Cy7-conjµgated fixable viability dye eFluor780 (Invitrogen). After washing, cells were suspended in sorting buffer for FACS analysis. Data analysis was performed using BD Verse software.

### Multiplexed cytokines measurement

The ProcartaPlex™ Multiplex Immunoassay (EPXR360-26092-901, Invitrogen) was used for measuring cytokine and chemokine levels in the mouse serum after infection. The test was performed according to the manufacturer’s instructions

### Histopathology

Livers and spleen from infected mice were removed and fixed in formalin overnight before embedding in paraffin. Slides were stained with hematoxylin and eosin, and the histopathology of tissues was analyzed using microscope.

### Confocal microscopy

The cells in PLF of infected mice were collected and pipetted into the wells of Lab-Tek Chamber Slides (ThermoFisher Scientific). After a 5 min incubation, detached cells were removed from each well. The cells were incubated with 4% paraformaldehyde for 10 min at room temperature, then permeabilized (0.25% Triton X-100) and blocked (1% BSA, 1 h, RT). Cells were stained with anti-mouse elastase (Bioss, dilution 1:200) and detected with Alexafluor 488-labeled secondary goat-anti-rabbit Fab antibody fragments (Life Technologies, dilution 1:1000). The slides were counterstained with DAPI by Prolong Diamond mounting medium (Invitrogen). Analysis was performed by confocal microscopy (Nikon Eclipse Ti).

### Isolation of neutrophils from peritoneal fluids

Cell isolation and identification were conducted based on previous research (17). In brief, mice were injected with 1.0 ml casein solution into the peritoneal cavity, and a second injection was performed after one night. The peritoneal fluid was collected 3 h after the second injection. Peritoneal exudate cells were mixed with 9 ml of Percoll gradient solution in a 10-ml ultracentrifuge tube. After ultracentrifuging (20 min at 60,650 × g 4°C), neutrophils in the second opaque layer were collected. Cell purity and viability were determined by FACS.

### Bactericidal activity of neutrophils

Neutrophils (2×10^6^ cells) were incubated with *S. suis* (MOI=5) for 1 or 3 h at 37˚C. A portion of the supernatants were taken and cultured overnight on THB plates at certain points to quantify non-engulfed extracellular bacteria. Part of the mixture was washed to remove the extracellular non-engulfed *S. suis*, then cells were laid onto 3 microscopic slides. Liu’s Stain was used to visualize cell morphology and 200 neutrophils per slide were counted for bacterial clearance rate. Bactericidal activity of neutrophils was determined by counting the number of live extracellular bacterial colonies, and the bacterial clearance rate was determined using Romanowsky Stain. Three replicate experiments were done for each experiment.

### Statistical analysis

The data in this study were statistically analyzed by GraphPad Prism 8 software and all data were present as the mean ± standard deviation (mean ± SD) unless otherwise noted. A two-way analysis of variance was used for the clinical scoring of mice. For the survival experiment, data was analyzed using the Kaplan-Meier method and Log-Rank test. For bacterial load, cytokine expression, and immune cells detection, data were analyzed with the Mann-Whitney U test; *P <*0.05, was considered significant.

## Results

### Fpr2 contribute to the pathogenesis of STSLS

**Figure 1:**
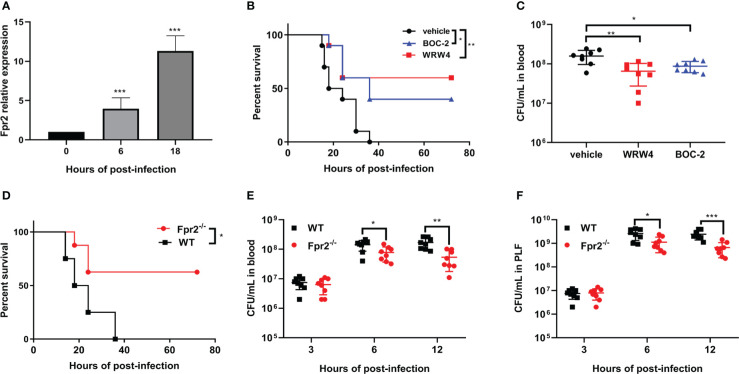
Fpr2 makes mice more susceptible to STSLS. **(A)** Wild-type mice were inoculated with standard *S. suis* dose intraperitoneally and Fpr2 mRNA levels in the spleen were evaluated. **(B)** Kaplan–Meier curves (N=10) of mice treated with BOC-2 (600 ng/kg), WRW4 (1 mg/kg), or a vehicle solution (THB) at 1 h after infection with a high dose of *S. suis*. **(C)** Blood bacteria in mice inoculated with standard dose of *S. suis*. **(D)** In WT and Fpr2^-/-^ mice infected with high dose of *S. suis*, Kaplan–Meier curves (N=8) were monitored for 72 h. **(E)** Bacterial loads in blood and **(F)** peritoneal lavage fluid was monitored at the indicated time points in mice infected with a standard dose of *S. suis.* *, *P* < 0.05; **, *P* < 0.01; ***, *P* < 0.001.

**Figure 2:**
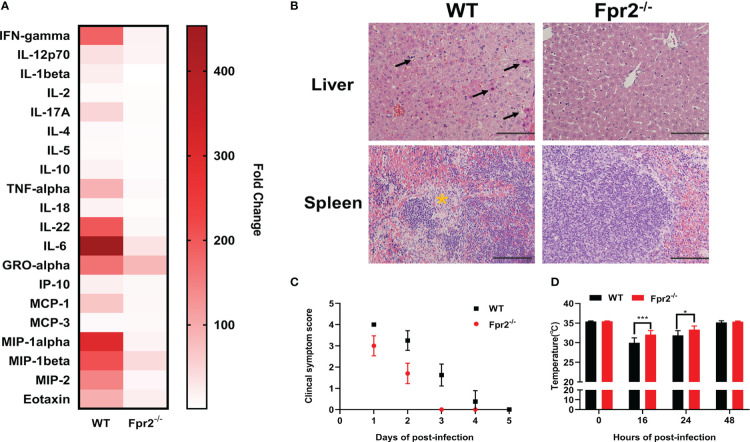
Fpr2 contributes to the induction of inflammation in STSLS. Wild-type and Fpr2^-/-^ mice were inoculated with a standard bacterial dose of *S. suis* intraperitoneally. **(A)** Serum was collected to detect inflammatory mediators by Luminex Assay at 12 h (N=4). **(B)** Mouse tissues were extracted for histological analysis. Arrows indicate apoptosis of hepatocytes. The yellow star indicates necrosis of Splenocyte. Size bars represent 100 μm. In clinical the observation experiment, **(C)** clinical scores and **(D)** anal rectal temperature were performed according to the criteria (N=10). *, *P* < 0.05; ***, *P* < 0.001.

To analyze the role of Fpr2 in the pathogenesis of STSLS, the expression of Fpr2 was detected in mice inoculated with *S. suis* intraperitoneally. Survival was monitored in WRW4 and BOC-2 (Fpr2 antagonist) intervention experiments. The increased expression of Fpr2 gene in the spleen implied a participatory role of Fpr2 during infection (**Figure 1A**). In the intervention trial, inhibiting Fpr2 with an antagonist significantly reduced the bacterial load and mortality of mice with STSLS (**Figures 1B, C**). Fpr2^-/-^ mice were used for a more detailed study of *S. suis* infection. After monitoring the bacterial load and mortality in WT and Fpr2^-/-^ STSLS mice, lower mortality rates and bacterial load were observed in the Fpr2^-/-^ mice (**Figures 1D-F**). A significant difference in bacterial load was observed in 6 h and 12 h post-infection both in blood and PLF (**Figures 1E, F**). These results indicate that Fpr2 exacerbates the acute symptoms of STSLS.Cytokine production, pathological changes, body temperature changes, and clinical evaluation were also monitored in infected mice. Mice deficient in Fpr2 had a lower inflammatory response supported by decreased soluble mediators and alleviated tissue injury at 12 h post-infection (**Figures 2A, B**). The soluble mediators of PLF in infected mice at 3 and 6 h were also detected, the deceased cytokines were significantly observed at 6 h in Fpr2^-/-^ mice, especially in CXCL1, CXCL2 and IL-6 (**Figure S1**). Hepatocyte apoptosis and splenocyte necrosis were only observed in WT mice (**Figure 2B**). Moreover, in contrast with Fpr2^-/-^ mice, induction of STSLS yielded worse clinical scores and prolonged hypothermia for WT mice. (**Figures 2C, D**). Collectively, these data indicate a potential contributing role of Fpr2 in STSLS pathogenesis.

### Fpr2 deficiency enhances neutrophil recruitment during the early stages of STSLS

**Figure 3:**
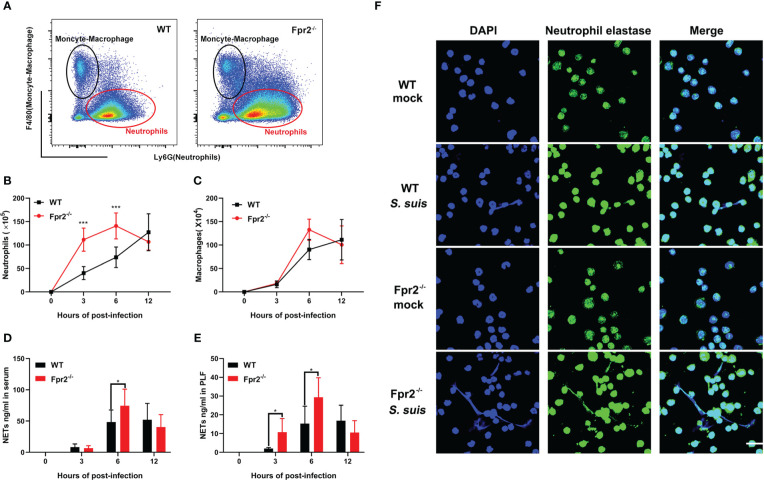
Fpr2 is involved in neutrophil migration. Wild-type and Fpr2^-/-^ mice were inoculated with a standard bacterial dose of *S. suis* intraperitoneally. **(A)** Scattergrams of neutrophils (identified as Ly6G^+^ F4/80^–^) and monocyte–macrophage (identified as Ly6G^–^F4/80^+^) positive events in peritoneal lavage fluids (PLF) from infected mice by flow cytometry analysis. **(B)** Absolute number of neutrophils or **(C)** macrophages/monocytes in PLF after infection at the indicated timepoint (N≥3). **(D)** NETs-detection in the serum or **(E)** PLF at the indicated timepoint (N≥3). **(F)** PLF of mice at 6 h post-infection visualized by immunofluorescence against neutrophil elastase (green) and DNA (blue). Size bars represent 10 μm. *, *P* < 0.05; ***, *P* < 0.001.

We focused on leukocyte activity during infection to understand the role of Fpr2 in the STSLS-induced inflammatory response. WT and Fpr2^-/-^ mice were inoculated with standard dose of *S. suis* (2 ×10^7^ CFU) intraperitoneally. Counts of leukocyte in the PLF were determined during infection. Macrophages/monocytes and neutrophils are the two main immune cells in the PLF after infection (**Figure 3A**). Flow cytometry analysis revealed striking neutrophilia in Fpr2^-/-^ mice at 3 and 6 h (**Figure 3B**). No significant difference was observed in macrophage counts between the two mice genotypes (**Figure 3C**). Higher level of NETs was also found in the plasma and PLF of Fpr2^-/-^ mice at 6 h post-infection (**Figures 3D, E**), which was further confirmed by immunofluorescence results. The filamentous chromatin fibers, areas of decondensed extracellular thread-like DNA colocalizing with neutrophil elastase, as visualized by confocal microscopy depict the NETs. More NETs construction was observed in the PLF of Fpr2^-/-^ mice (**Figure 3F**). Together, these data suggest that Fpr2 impaired the recruitment of neutrophils and production of NETs during STSLS.

### Fpr2 has no direct impact on NETs construction

**Figure 4:**
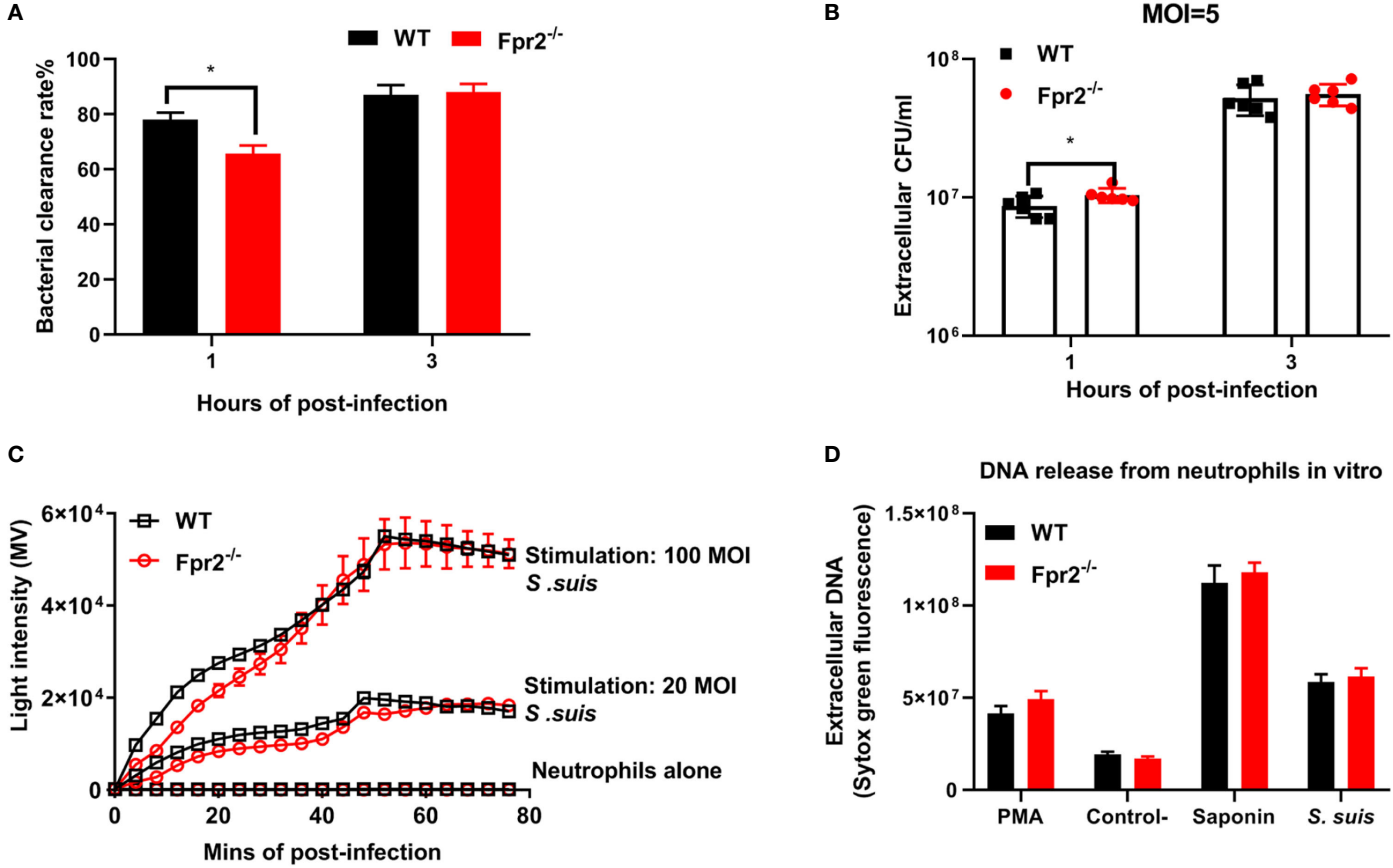
Fpr2 has no direct impact on NETs construction *in vitro*. WT and Fpr2 neutrophils stimulated with *S. suis* (MOI = 5) *in vitro*. **(A)** Bacterial clearance rate and **(B)** extracellular bacteria were monitored at 1 and 3 h. **(C)** In the ROS production experiment, the level of light intensity was measured when neutrophils were stimulated with different MOIs of *S. suis*. **(D)** The level of NETs produced by WT and Fpr2^-/-^ neutrophils (MOI =100) was evaluated at 3 h post-infection. PMA was used as a positive control. Saponin was used to calibrate the number of cells. *, *P* < 0.05.

Having established active neutrophil recruitment and higher levels of NETs structure in Fpr2^-/-^ mice suffering from STSLS, we further explored how Fpr2 affects the morphology or function of neutrophils. The bactericidal ability and oxidative stress properties of WT and Fpr2^-/-^ neutrophils were evaluated after cells were treated by *S. suis* (MOI=5) *in vitro*. To determine bactericidal ability, the bacterial clearance rate was evaluated at 1 and 3 h (**Figure 4A**). The extracellular bacterial load, which reflects the bactericidal capacity of WT and Fpr2^-/-^ neutrophils, was also evaluated (**Figure 4B**). Bacterial clearance rate calculation of stimulated neutrophils revealed that Fpr2^-/-^ cells exhibited lower bacterial clearance rate than WT mice at 1 h post treatment. However, this had no influence on the bactericidal ability of neutrophils, as the phagocytic bacteria and extracellular bacteria between the cells of the two genotypes showed no significant differences at 3 h. Regarding oxidative stress properties, ROS production was monitored when WT and Fpr2^-/-^ cells were treated with *S. suis* at different MOIs. Although there was higher light intensity in the WT group before 25 min, as the infection progressed no differences were observed between the cells of the two genotypes at 40 min (**Figure 4C**). No significant difference was observed in the NETs production experiment when the two genotype neutrophils were treated with *S. suis* for 3 h *in vitro*. (**Figure 4D**). These results indicate that Fpr2 did not directly affect ROS and NETs production of neutrophils *in vitro*, and that the improved leukocyte infiltration of Fpr2 deficient mice may account for the higher levels of NETs observed *in vivo*.

### LXA4 and AnxA1 promote bacterial proliferation through Fpr2

**Figure 5:**
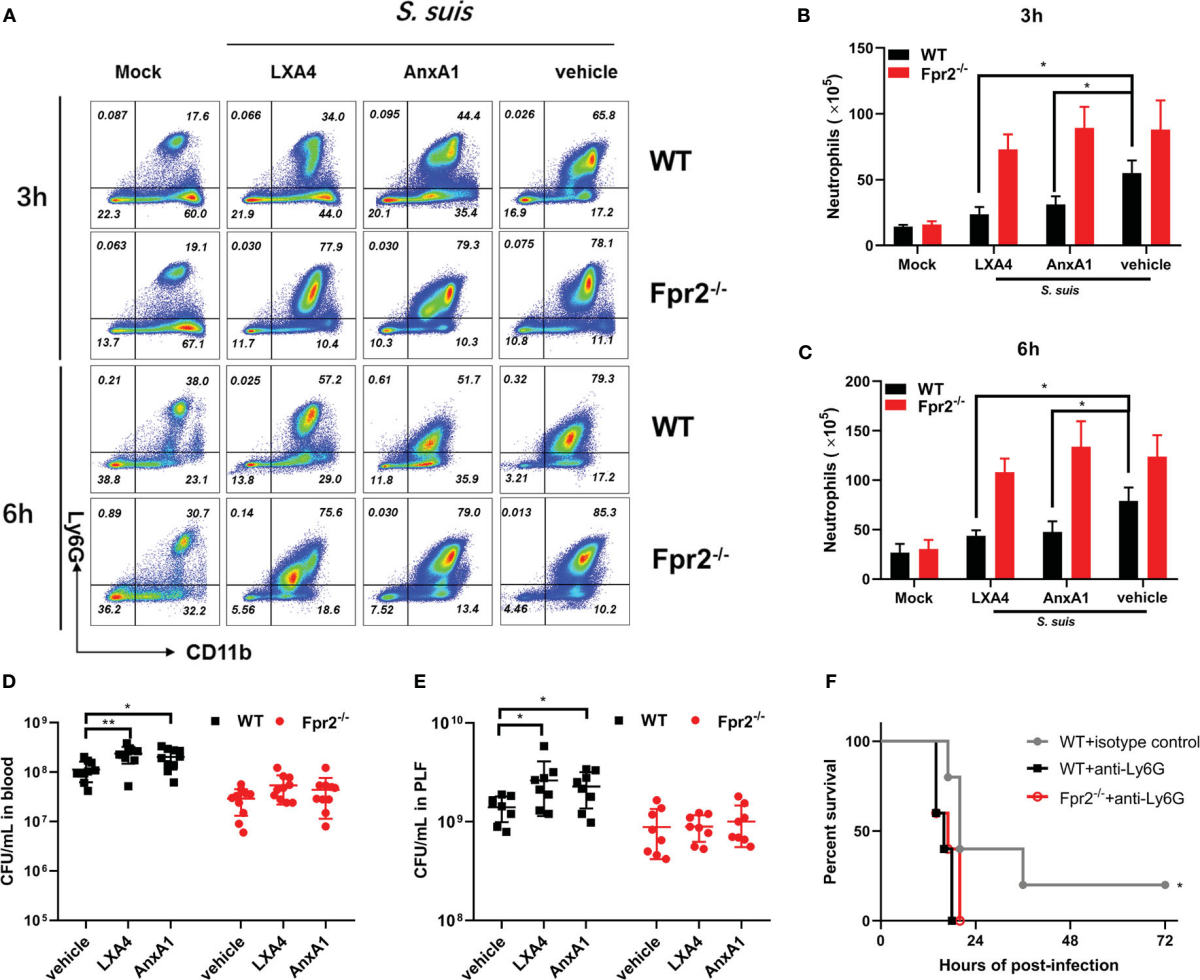
Fpr2 involvement in LXA4 or AnxA1-mediated anti-recruitment function. LXA4 or AnxA1 were used to intervene in STSLS in WT and Fpr2^-/-^ mice. **(A)** Scattergrams of neutrophils (identified as Ly6G^+^ CD11b^+^) positive events in PLF from WT and Fpr2^-/-^ mice by flow cytometry analysis. Count of neutrophils in the PLF of WT and Fpr2^-/-^ mice at **(B)** 3 h or **(C)** 6 h was evaluated (N≥3). Bacterial load in **(D)** blood or **(E)** PLF at 6 h was monitored (N≥8). **(F)** Survival of mice pretreated with anti-Ly6G antibody or isotype before infection (N=5). *, *P* < 0.05; **, *P* < 0.01.

Administration of LXA4 or AnxA1 efficiently alleviates neutrophil accumulation, reduces inflammation, and attenuates many diseases. Our previous study elucidated the potential therapeutic effect of AnxA1 in *S. suis* meningitis. In this study, LXA4 and AnxA1 were used as an intervention in STSLS infection to explore the potential effects of LXA4 and AnxA1 in STSLS treatment. Analysis of cytometer results showed the potential anti-recruitment function of LXA4 and AnxA1 in WT mice at 3 and 6 h post-infection, although no effect was observed in Fpr2^-/-^ mice. (**Figures 5A-C**). The bacterial load in the blood and PLF at 6 h was monitored. High bacterial loads were observed in LXA4 or AnxA1 treated WT mice, but this effect was not observed in Fpr2^-/-^ mice (**Figures 5D, E**). In order to confirm that Fpr2 primarily affects the activity of neutrophils in STSLS, we used anti-Ly6G antibody to block the neutrophils. Treatment with anti-Ly6G eliminated observed differences between the WT and Fpr2^-/-^ mice (**Figure 5F**). Compared to the isotype group, pretreatment with anti-Ly6G increased mortality and accelerated death from STSLS. Neutrophil recruitment during the early stages of STSLS plays an indispensable role in bacterial clearance, which is a key step regulated by Fpr2 during the inflammation response.

**Figure 6:**
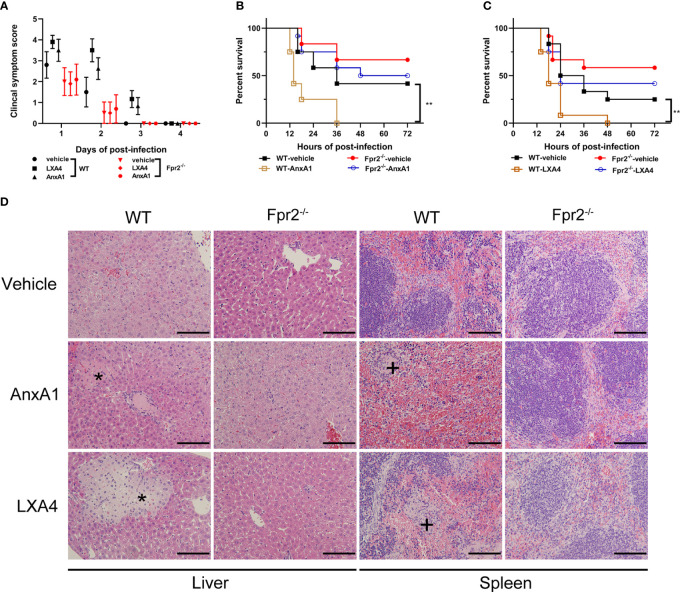
Down-regulation of recruitment by Fpr2 aggravate STSLS. LXA4 or AnxA1 were used to intervene in *S. suis* infection (3×10^7^ CFU) of WT and Fpr2^-/-^ mice. **(A)** Clinical symptom scores were evaluated (N=10). In survival experiments, **(B)** AnxA1, **(C)** LXA4, or a vehicle solution (THB) were used as pre-treatment in mice, then the mortality was recorded within 72 h (N=12). **(D)** Histopathological analysis was also performed (N=3). The asterisk (*) and plus (+) indicates histopathological lesion in the liver or spleen, respectively. Size bars represent 500 µm. **, *P* < 0.01.

Clinical symptoms score, lethality test and histopathology changes in STSLS, were also assessed when LXA4 and AnxA1 were used. Treatment with LXA4 or AnxA1 in WT mice increased the clinical symptom score and lethality in these mice, indicating a detrimental role of anti-recruitment drugs in STSLS (**Figures 6A-C**). Exaggerated tissue damage was also observed in WT but not Fpr2^-/-^ mice (**Figure 6D**). Those data further demonstrate that neutrophil recruitment during the early stages of infection plays a crucial role in controlling bacterial proliferation and dissemination. The anti-recruitment mediators LXA4 and AnxA1 aggravated tissue damage and increased the inflammatory response in STSLS *via* Fpr2.

### Bidirectional effects of NETs on *Streptococcus suis* proliferation and host protection

**Figure 7:**
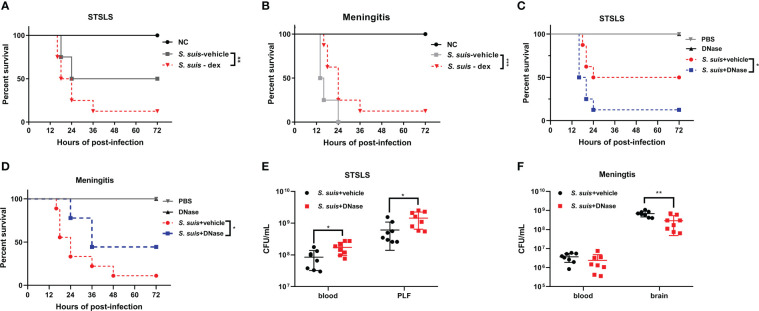
Bidirectional effects of recruitment in different *S. suis* infection pathway models. Kaplan–Meier curves of Fpr2^-/-^ mice intervened with Dexamethasone in **(A)** STSLS or **(B)** *S. suis* meningitis (N=8). Kaplan–Meier curves of WT mice intervened with DNase in **(C)** STSLS or **(D)** meningitis model (N=8). Bacterial load of WT mice intervened with DNase in **(E)** STSLS or **(F)** meningitis model (N=8). *, *P* < 0.05; **, *P* < 0.01.

Previous studies from our laboratory have reported the beneficial role of Fpr2 in bacterial clearance and inflammation resolution during *S. suis* meningitis. Fpr2^-/-^ mice were highly susceptible to *S. suis* meningitis, and displayed increased bacterial dissemination and neutrophil migration. AnxA1 attenuated inflammatory responses and neutrophil invasion through Fpr2 during *S. suis* meningitis. When compared with the current study on the STSLS model, both studies revealed an anti-recruitment function of Fpr2 during infection, but opposite effects on disease development in the different infection models. We speculated that neutrophil recruitment may play a different role in bacterial dissemination and disease treatment between meningitis and STSLS. Dexamethasone (dex) is a powerful anti-inflammatory compound inhibiting inflammatory cell recruitment and production of proinflammatory cytokines. Thus, dexamethasone was used to pretreat mice suffering from *S. suis* induced STSLS or meningitis to investigate the potential role of early inflammation at different infection sites. Injection of dexamethasone decreased the mortality of Fpr2^-/-^ mice in the meningitis model, but accelerated the death in Fpr2^-/-^ mice with STSLS (**Figures 7A, B**). Similar results were also obtained in WT mice (**Figure S2**). These results indicate that inflammation during the early stages of disease development is detrimental to host survival in the meningitis model, but beneficial to survival in the STSLS model. This finding was aligned with the results from a DNA intervention experiment which clarified the dual role of NETs during STSLS and meningitis. DNase I (DNase), which effectively degrades NET-associated DNA, was infused at the time of infection to prevent the accumulation of NETs in both the meningitis and STSLS models. Survival and bacterial load of infected mice in both infected models were evaluated. DNase significantly increased the mortality of the STSLS mice, but reduced the mortality of the meningitis mice (**Figures 7C, D**). Additionally, DNase treatment significantly aggrandized the bacterial load in the STSLS mice, but had an opposite effect on the meningitis group (**Figures 7E, F**). This suggests that NETs play protective roles in bacterial cleaning and host individual survival in the initial stages of STSLS, whereas it interferes with bacterial clearance in the CNS and aggravates the severity of meningitis. These results highlight a dual role of neutrophils and NETs on *S. suis* proliferation and host protection in meningitis and STSLS models.

## Discussion

The present study aimed to investigate the role of Fpr2 in the pathogenesis of the disease Streptococcal toxic shock-like syndrome (STSLS) and to explore the potential effects of the pro-resolution agents LXA4 or AnxA1 in disease treatment. The results demonstrated a detrimental impact of Fpr2 on STSLS pathogenesis by restricting the recruitment of neutrophils to the infection site, especially in the early stages of infection. Interference with Fpr2 or neutrophil recruitment activity may be a new therapeutic strategy to treat STSLS. Interestingly, a completely opposite effect of early neutrophil activity was observed in the *S. suis* meningitis models. Their powerful bactericidal and oxidative stress functions make neutrophils the most essential immune cells in the host defense system during infectious disease progression. However, neutrophils may exert opposite effects on disease progression in systemic or local infections in the early or late stages. The dual role of NETs may hinder bacterial clearance in the CNS, but accelerate bacterial clearance in the STSLS model. These findings indicate that regulating neutrophil recruitment is a potential treatment strategy to control infectious disease.

The therapeutic and anti-inflammatory effects of LXA4 have been demonstrated in many studies. During inflammatory brain injury after intracerebral hemorrhage, LxA4 reduced brain-infiltration by neutrophils and ameliorated the inflammatory brain injury (18). The administration of LXA4 has also been reported to suppress inflammation by and infiltration of neutrophils in experimental subarachnoid hemorrhage rat models (19). Interestingly, LXA4 exhibits a dual role in *Klebsiella pneumoniae* induced sepsis. Treatment with LXA4 worsened the infection and decreased cell migration in early sepsis but improved the survival rate by reducing the excessive inflammatory response in late sepsis (15). These findings are highly consistent with our results. Fpr2^-/-^ mice have been used in many studies focused on infection or inflammation. Previous studies have confirmed that the antimigratory compounds lipoxin A and annexin A1 were reduced notably in Fpr2^-/-^ mice (20). In the *Streptococcus pneumoniae* (*S. pneumoniae*) infected mouse model, increased infiltration made Fpr2^-/-^ mice highly susceptible to *S. pneumoniae* meningitis (21). A study of ageing Fpr2^-/-^ mice showed an integrative role of Fpr2 in cardiac inflammation-resolution processes and obesogenic aging. Fpr2 dysfunction magnified obesogenic cardiomyopathy and neutrophil recruitment in aging mice (22). These studies highlight the essential regulatory roles of Fpr2 in different inflammatory responses.

Fpr2 performs both pro-inflammatory and pro-resolution immune functions depending on the presence of diverse ligands. Mice deficient in Fpr2 experienced more severity *Listeria* or *S. aureus* infection due to decreased leukocyte recruitment (23). This may be due to in the differing effects of Fpr2 on localized and systemic infections. Another potential explanation is the variable by-products generated during infection by different pathogens, as one virulence-associated factor of *S. suis*, Suilysin was associated with bacterial aggressiveness during infection by increasing cell-damaging effects. Formyl peptides of mitochondria, when released in response to cell damage, can be directly recognized by Fpr2 receptors and trigger an intense inflammatory response (24, 25).

The CNS has a poorly developed lymphatic drainage system and unique composition of the capillaries compared to most other organs (26). Since *Foldi* first proposed a role for the lymphatic system in the CNS and *Louveau* made the official discovery of it *in vivo* (27)much more about these processes are understood, but there are still plenty of mysteries surrounding the lymphatic network of the CNS. The unique composition of the capillaries in the brain, such as the end feet of astrocytes and pericytes, also results in different functions and behaviors. Strong neutrophil recruitment could potentially destroy normal tissue, interfere with cerebrospinal fluid circulation, and hinder bacterial clearance.

The protective function of NETs-including how they contribute to bacterial removal and inflammation resolution-in the initial stages of sepsis have been described for many infectious diseases, including those resulting from *S. suis*, *S. pneumoniae*, and *S. aureus* infections (28–30). A study on protein arginine deiminase 4 knockout mice demonstrated that killing of *Shigella flexneri* is mediated by NETs (31). However, detrimental effects of NETs in sepsis have also been described, as excessive NETs formation was associated with multiple organ injuries, a hyperinflammatory response, and thrombosis (32). Aberrant amounts of NETs could occlude capillaries, impair microcirculation, and injure normal tissues (33). Many pathogens have distinct DNases to degrade the DNA of NETs. Both endonuclease A (designated EndAsuis) and secreted *S. suis* nuclease A are capable of degrading NETs *in vitro* (34). Nuclease expression by *S. aureus* was associated with delayed bacterial clearance and facilitated bacterial escape from NETs (35). Hosts have also evolved unique tools, such as the antimicrobial peptide LL37, to protect against degradation by bacterial nucleases (36). A previous study revealed that Fpr2^-/-^ mice had excessive NETs formation after bacterial infection and that the lipoxin pathway could be a potent modulator (37). The involvement of Fpr2 in NETosis-related pathways may result from modifying calcium flux, which effectively promotes neutrophil apoptosis (33).

Fpr2 primarily causes increased neutrophil recruitment in *S. suis* infection, which exerts different effects on disease progression and host protection. Therapies that inhibit the synthesis or action of LXA4 and/or AnxA1, or that stimulate neutrophil activation could be useful for STSLS treatment. We also emphasize the delicate balance of inflammation response, which should be regulated under precise control with relation to different infection stages and sites. Therapy targeting Fpr2 or recruitment could potentially be developed as an additional treatment to antibiotics for infectious diseases.

## Data availability statement

The original contributions presented in the study are included in the article/**Supplementary Material**. Further inquiries can be directed to the corresponding authors.

## Ethics statement

The animal study was reviewed and approved by Animal Center of the Academy of Military Medical Sciences.

## Author contributions

CN, SG and XL performed the whole experiment and formal analysis. CN and XL wrote the original draft preparation. YJ, ZM and DK designed the study and revised the manuscript. All authors contributed to the article and approved the submitted version.

## Supplementary material

Supplementary Figure 1 Wild-type and Fpr2^-/-^ mice were inoculated with a standard bacterial dose of *S. suis* intraperitoneally. PLF was collected to detect inflammatory mediators **(A)** CXCL1, **(B)** CXCL2 and **(C)** IL-6 by ELISA at 3 and 6 h (N=4). ***, *P* < 0.001.

Supplementary Figure 2 Kaplan–Meier curves of Wild-type mice intervened with Dexamethasone in **(A)** STSLS or **(B)** *S. suis* meningitis (N=5). *, *P* < 0.05.
