# Supplementary figures and images for: Fpr2 exacerbates Streptococcus suis-induced streptococcal toxic shock-like syndrome via attenuation of neutrophil recruitment
Source: Front Immunol. 2023 Jan 27;14:1094331. doi: 10.3389/fimmu.2023.1094331 (PMC9911822; doi:10.3389/fimmu.2023.1094331)

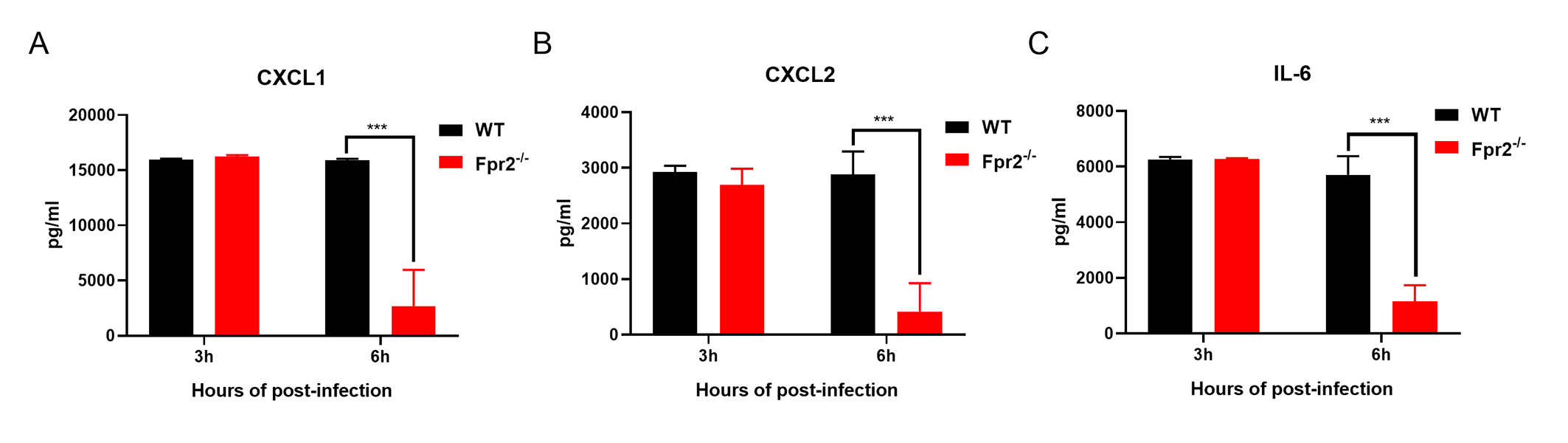

Supplement: Supplementary Figure 1 — Wild-type and Fpr2-/- mice were inoculated with a standard bacterial dose of S. suis intraperitoneally. PLF was collected to detect inflammatory mediators (A) CXCL1, (B) CXCL2 and (C) IL-6 by ELISA at 3 and 6 h (N=4). ***, P < 0.001. [file Image_1.tif]

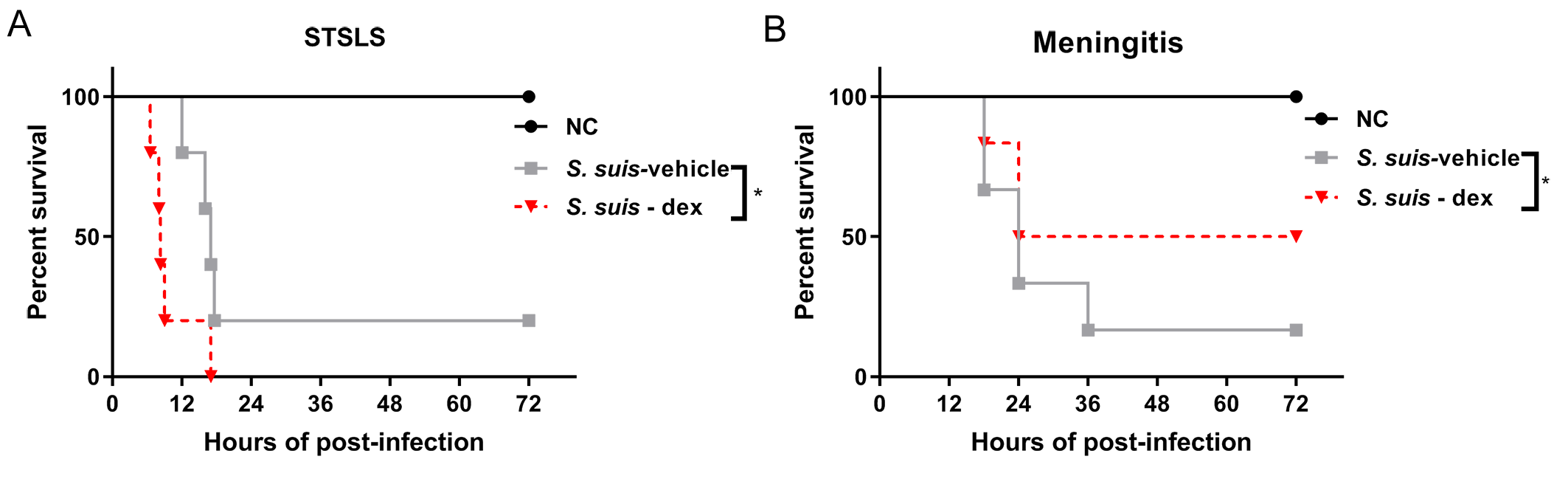

Supplement: Supplementary Figure 2 — Kaplan–Meier curves of Wild-type mice intervened with Dexamethasone in (A) STSLS or (B) S. suis meningitis (N=5). *, P < 0.05. [file Image_2.tif]
